# Effect of xenon on brain injury, neurological outcome, and survival in patients after aneurysmal subarachnoid hemorrhage—study protocol for a randomized clinical trial

**DOI:** 10.1186/s13063-023-07432-8

**Published:** 2023-06-19

**Authors:** Mikael Laaksonen, Jaakko Rinne, Melissa Rahi, Jussi P. Posti, Ruut Laitio, Juri Kivelev, Ilkka Saarenpää, Dan Laukka, Juhana Frösen, Antti Ronkainen, Stepani Bendel, Jaakko Långsjö, Marika Ala-Peijari, Jani Saunavaara, Riitta Parkkola, Mikko Nyman, Ilkka K. Martikainen, Alex M. Dickens, Juha Rinne, Mika Valtonen, Teijo I. Saari, Timo Koivisto, Paula Bendel, Timo Roine, Antti Saraste, Tero Vahlberg, Juha Tanttari, Timo Laitio

**Affiliations:** 1 Department of Perioperative Services, Intensive Care Medicine and Pain Management, Turku University Hospital and University of Turku, P.O. Box 52, FIN-20521 Turku, Finland; 2 Neurocenter, Department of Neurosurgery and Turku Brain Injury Center, Turku University Hospital and University of Turku, Turku, Finland; 3 Department of Neurosurgery, Faculty of Medicine and Health Technology, Tampere University Hospital, University of Tampere, Tampere, Finland; 4 Department of Intensive Care, Kuopio University Hospital, University of Eastern Finland, Kuopio, Finland; 5 Department of Anesthesiology and Intensive Care, Tampere University Hospital and University of Tampere, Tampere, Finland; 6 Department of Medical Physics, Turku University Hospital and University of Turku, Turku, Finland; 7 Department of Radiology, Turku University Hospital and University of Turku, Turku, Finland; 8 Department of Radiology, Tampere University Hospital and University of Tampere, Tampere, Finland; 9 Analysis of the metabolomics, University of Turku, Turku BioscienceTurku, Finland; 10 Turku PET Centre, Turku University Hospital and University of Turku, Turku, Finland; 11 Department of Neurosurgery, Kuopio University Hospital, University of Eastern Finland, NeurocenterKuopio, Finland; 12 Department of Radiology, Kuopio University Hospital, Kuopio, Finland; 13 Department of Neuroscience and Biomedical Engineering, Aalto University School of Science, Espoo, Finland; 14 Heart Centre, Turku University Hospital, Turku University Hospital and University of Turku, Turku, Finland; 15 Department of Biostatistics, University of Turku, Turku, Finland; 16 Technical Analysis, Elomatic Consulting & Engineering, Thane, India

**Keywords:** Xenon, Neuroprotection, Aneurysmal subarachnoid hemorrhage, aSAH, Randomized controlled trial

## Abstract

**Background:**

Aneurysmal subarachnoid hemorrhage (aSAH) is a neurological emergency, affecting a younger population than individuals experiencing an ischemic stroke; aSAH is associated with a high risk of mortality and permanent disability. The noble gas xenon has been shown to possess neuroprotective properties as demonstrated in numerous preclinical animal studies. In addition, a recent study demonstrated that xenon could attenuate a white matter injury after out-of-hospital cardiac arrest.

**Methods:**

The study is a prospective, multicenter phase II clinical drug trial. The study design is a single-blind, prospective superiority randomized two-armed parallel follow-up study. The primary objective of the study is to explore the potential neuroprotective effects of inhaled xenon, when administered within 6 h after the onset of symptoms of aSAH. The primary endpoint is the extent of the global white matter injury assessed with magnetic resonance diffusion tensor imaging of the brain.

**Discussion:**

Despite improvements in medical technology and advancements in medical science, aSAH mortality and disability rates have remained nearly unchanged for the past 10 years. Therefore, new neuroprotective strategies to attenuate the early and delayed brain injuries after aSAH are needed to reduce morbidity and mortality.

**Trial registration:**

ClinicalTrials.gov NCT04696523. Registered on 6 January 2021.

EudraCT, EudraCT Number: 2019-001542-17. Registered on 8 July 2020.

## Administrative information

Note: the numbers in curly brackets in this protocol refer to SPIRIT checklist item numbers. The order of the items has been modified to group similar items (see http://www.equator-network.org/reporting-guidelines/spirit-2013-statement-defining-standard-protocol-items-for-clinical-trials/).

**Table:** 

Title {1}	Effect of xenon on brain injury, neurological outcome, and survival in patients after aneurysmal subarachnoid hemorrhage (Xe-SAH) - A randomized clinical trial
Trial registration {2a and 2b}.	ClinicalTrials.gov, Identifier: NCT04696523. Registered 6th January 2021 EudraCT, EudraCT Number: 2019-001542-17. Registered 8th July 2020
Protocol version {3}	1.1.2022 - Version 1.2
Funding {4}	TL: State Research Funding - Academy of Finland. ML: personal research grants: the Finnish Medical Foundation, Finnish Society of Anaesthesiologists.
Author details {5a}	1. Department of Perioperative Services, Intensive Care Medicine and Pain Management, Turku University Hospital and University of Turku, Finland 2. Neurocenter, Department of Neurosurgery and Turku Brain Injury Center, Turku University Hospital and University of Turku, Finland. 3. Department of Neurosurgery, Tampere University Hospital and faculty of Medicine and Health Technology, University of Tampere, Finland. 4. Department of Anesthesiology and Intensive Care, Tampere University Hospital and University of Tampere, Finland. 5. Department of Intensive Care, Kuopio University Hospital and University of Eastern Finland. 6. Department of Medical Physics, Turku University Hospital and University of Turku, Finland. 7. Department of Radiology, Turku University Hospital and University of Turku, Finland. 8. Department of Radiology, Tampere University Hospital and University of Tampere, Finland. 9. University of Turku, Turku Bioscience, Analysis of the metabolomics. Turku, Finland. 10. Turku PET Centre, Turku University Hospital and University of Turku. Finland. 11. Department of Neurosurgery, Neurocenter, Kuopio University Hospital and University of Eastern Finland. 12. Department of Biostatistics, University of Turku, Finland. 13. Department of Radiology, Kuopio University Hospital, Finland. 14. Department of Neuroscience and Biomedical Engineering, Aalto University School of Science, Helsinki, Finland. 15. Heart Centre, Turku University Hospital; Turku University Hospital and University of Turku, Finland. 16. Technical Analysis, Elomatic Consulting&Engineering, Finland.
Name and contact information for the trial sponsor {5b}	An investigator-initiated clinical drug study Laitio, T (Principal Investigator) timo.laitio@elisanet.fi
Role of sponsor {5c}	This is an investigator-initiated clinical trial. Therefore, the funders played no role in the design of the study and collection, analysis, and interpretation of data and in writing the manuscript.

## Introduction

### Background and rationale {6a}

Aneurysmal subarachnoid hemorrhage (aSAH) is a neurological emergency which is associated with a high risk of mortality and permanent disability. Subarachnoid hemorrhages account for approximately 5–10% of all strokes; however, the case mortality rate of aSAH at 1 year is considerably higher (50%) when compared to ischemic stroke. Up to 10–15% of patients with aSAH die before reaching the hospital [1–7]. In addition to mortality, aSAH is associated with a substantial impairment in the quality of life, with significant neurological deficits and psychological sequelae. About one in five survivors of aSAH will not regain functional independence and only every fourth regains the same level of functionality as before the aSAH [1–6]. In worldwide estimates, when compared with ischemic strokes, aSAH patients are disproportionately younger, i.e., of working age with the age at insult mostly below 66 years. In Finland, the mean age of the event is as young as 55 years [8]. Subsequently, aSAH is associated with a considerable loss of independent functionality and responsible for the development of dependency early in life. Novel treatments are needed to mitigate the effects of aSAH both in the immediate aftermath (early brain injury, EBI) and thereafter to improve the current dismal outcomes.

EBI is a major cause of mortality in aSAH patients [9–11]; it is defined as the period within the first 72 h after the ictus and describes the immediate overall brain injury after the aSAH. The initial clinical severity of the aSAH is the most important factor determining the overall outcome; this is assessed by a preliminary assessment of radiographic findings and neurological consciousness using the Glasgow Coma Scale (GCS), together with Hunt-Hess or WFNS grading. Initial radiographic markers of EBI (e.g., cytotoxic edema) are significant predictors of secondary complications and outcomes [9–11]. The pathophysiological disturbances underlying the radiographic findings during the EBI period are not completely understood; however, acute global ischemia, ischemia/reperfusion injury from the transient circulatory arrest at ictus, increased intracranial pressure (ICP), decrease in cerebral blood flow (CBF), and perfusion pressure (CPP) are clinically relevant pathological processes which can result in neuronal death. In addition, other pathophysiological mechanisms during the EBI period include disruptions of the blood-brain barrier, brain swelling, vasogenic and cytotoxic edema, acute vasospasm, dysfunction of autoregulation, altered ionic homeostasis, disruption of vascular integrity, excitotoxicity (e.g., via glutamate-activated receptors), thrombin activation, oxidative stress, inflammation (via activation of microglial cells), increased levels of matrix metalloproteinase (MMP) 9, and activation of the nitric oxide synthase (NOS) pathway. It has been suggested that there may be a causal relationship between delayed cerebral ischemia (DCI) and the severity of EBI. Therefore, an implementation of new treatment modalities targeting EBI seems to represent a promising avenue to attenuate its secondary injuries such as DCI and cerebral infarction [9–11].

Even after successful microsurgical or endovascular treatment of the ruptured aneurysm, DCI is still a potentially devastating clinical syndrome related to aSAH and it is the most significant cause for morbidity and mortality in patients who survive the initial aSAH [1–3, 12]. DCI is a clinical syndrome of focal neurological and cognitive deficits presenting in about 30% of aSAH patients at 3–14 days after the initial insult, which might progress to infarction [1–5, 12]. While an angiographically evident vasospasm in the cerebral arteries can be detected in 70% of patients, less than half of them develop any symptoms or ischemia in the territory supplied by the narrowed artery [1]. In addition, treatment with the calcium-channel blocker, nimodipine, which is currently the standard of care and the only pharmacological intervention that has been shown to reduce the risk of DCI and improve neurologic outcome by one-third after aSAH, does not alter the incidence or severity of delayed cerebral artery vasospasm [13]. Furthermore, other clinical trials involving drugs that exert a significant impact on vasospasm did not have any impact on the risk of DCI or outcomes [1, 12, 14]. Consequently, recent research has questioned whether there is truly a direct causal relationship between cerebral vasospasm and DCI and it has been suggested that DCI following an aSAH is actually the result of a variety of different pathophysiological mechanisms, which are currently incompletely understood [1, 12, 15, 16]. From preclinical and clinical studies, a number of possible mechanisms other than vasospasm to explain the link between DCI and infarction have been proposed including microthromboembolism, cortical spreading depolarization and ischemia, microcirculatory dysfunction, inflammation, and impaired cerebral autoregulation [12]. It has been recommended that the main outcomes of clinical trials investigating strategies to prevent DCI should measure the extent of cerebral infarction by either radiologic imaging or autopsy biopsy as well as the patient’s functional outcome [17].

The anesthetic properties of the noble gas xenon have been recognized since the late 1940s. Xenon is a near-perfect anesthetic gas as it is not metabolized, it is remarkably safe and efficient, has a minimal effect on hemodynamics, rapidly crossing the blood-brain barrier, as well as possessing very rapid induction and recovery characteristics due to its extremely low solubility (blood/gas partition coefficient of 0.115) [18, 19]. After the pivotal discovery that xenon is a non-competitive antagonist of the N-methyl-D-aspartate (NMDA) subtype of the glutamate receptor, numerous preclinical studies in three different species of animals and in various models of acute neuronal injury have confirmed that xenon confers neuroprotection [20]. There are many experimental models in which the beneficial effects of xenon have been evident, e.g., administration of excitotoxins or oxygen deprivation in rats [21–23], cardiopulmonary bypass (CBP) in rats [24], middle cerebral artery occlusion in mice [25, 26], cardiac arrest in pigs [27, 28], hypoxic-ischemic insult in rats [29, 30], TBI in mice [31–33], and SAH in rats. Neuroprotection has been demonstrated by a reduction in the infarct volume after focal ischemia [25, 26], attenuated short- and long-term neurologic and neurocognitive dysfunction [27, 28, 34–37], and a reduction in the extent of the neurohistopathological damage [20, 29, 31–37]. The neuroprotective properties of xenon were most clearly evident when the gas was administered either immediately or 2–6 h after injury [20, 30–37]. In addition to animal studies, the effect of xenon has been studied in humans mostly due to its cardioprotective properties. The potential cardioprotective properties of xenon anesthesia have been at least partly attributed to xenon’s ability to preserve cardiovascular stability by maintaining (i) systolic blood pressure, (ii) myocardial contractility, (iii) stroke volume, and (iv) preload, accompanied by an inotrope-sparing effect [38–40]. In addition, multiple molecular targets have been identified as being implicated in xenon’s cardioprotective conditioning effect. Phosphorylation of protein kinase cƐ, protein kinase B, and glycogen synthase kinase 3 β by xenon has been reported to inhibit the Ca2+-induced opening of transition pores in mitochondria, a process known to preserve mitochondrial function and prevent ischemic reperfusion injury and cell death [41]. Xenon’s potential cardioprotective properties were supported by the outcomes of Xe-Hypotheca trial; inhaled xenon reduced myocardial injury, as demonstrated by the significantly lower release of troponin-T from baseline to 72 h after cardiac arrest in the xenon-treated group as compared with the control group [40]. In a multicenter study on coronary artery bypass patients, the troponin I levels in the group who received xenon were significantly lower than the corresponding values in the sevoflurane group [42].

In 2016, we reported (Xe-HYPOTHECA trial; Clinicaltrials.gov NCT00879892) that xenon was able to attenuate the white matter injury after global ischemia after cardiac arrest [43]. The effect was mainly due to less extensive myelin damage in the xenon-treated group than in the control group. This was demonstrated by a significantly higher global fractional anisotropy of diffusion tensor imaging of white matter tracts in the xenon-treated patients than in the control patients [43]. The proper function of white matter is fundamental for good neurocognitive performance. Nonetheless, the Xe-HypotheCA trial was not powered to assess the clinical outcome. For example, the 6-month mortality was 15/55 (27.7 %) in the xenon-treated group and 19/55 (34.5 %) in the control group (adjusted HR 0.49 [95% CI 0.23–1.01], *P*=0.053). The interpretation of the authors was that this result was due to a combination of xenon’s neuro-and cardioprotective properties, which may also be clinically beneficial after aSAH.

Despite improvements in medical technology and advances in medical science, aSAH mortality and disability rates have remained virtually the same during the past 10 years [7]. The age and illness severity adjusted in-hospital mortality of 25–30% has also not changed over a span of 12 years, from 2003 to 2015 [5, 6]. Therefore, it is clear that new neuroprotective strategies are needed to attenuate the early and delayed brain injury occurring after an aSAH in order to improve the current dismal situation.

#### Objectives {7}

##### ***Hypothesis***

The primary hypothesis of the study is that the global white matter injury would be less severe as demonstrated with higher fractional anisotropy of DTI (diffusor tensor imaging) on the 1st MRI in patients treated with xenon, as compared to the patients who did not receive xenon.

#### Objectives

The primary objective is:

To explore whether xenon is neuroprotective in aSAH patients.

The secondary objectives are to explore the neuroprotective effects of xenon on:

1. The white matter injury in the cerebellum and corpus callosum. Recent studies have revealed that fractional anisotropy of white matter at 72 h post-SAH measured globally, in the cerebellum and in the corpus callosum, had an independent predictive value for both DCI and for a poor neurological outcome (i.e., mRS 3-6) [44].
2. The incidence and severity of EBI within 72 h after the appearance of SAH symptoms, DCI, and cerebral infarction (according to a consensus statement [17] and consensus guidelines [5]: (1) DCI is defined as a new focal neurological deficit (such as hemiparesis, aphasia, apraxia, hemianopia, or neglect) or a decrease of at least 2 points in the Glasgow Coma Scale (either in the total score or in one of its individual components). This should last for at least 1 h and not be due to other causes (e.g., hydrocephalus, seizures, metabolic derangement, infection, sedation). DCI is not apparent immediately after aneurysm occlusion and cannot be attributed to other causes by means of clinical assessment, CT or MRI scanning of the brain, and appropriate laboratory studies; (2) cerebral infarction is defined as a new infarct on follow-up imaging (i.e., in any of the following: 2nd MRI, CT, CTA, DSA, and perfusion CT) between day 4 and 6 weeks after the initial appearance of SAH symptoms.
3. The incidence of adverse events, serious adverse events, suspected unexpected serious adverse reactions
4. The inflammatory process, i.e., assessed by the extent of microglial activation and the levels of cytokine in cerebral spinal fluid (CSF)
5. The need and duration of intracerebral pressure (ICP) treatments
6. Plasma catecholamine levels
7. The release of troponin T
8. The release of neuron-specific enolase (NSE), neurofilament light (NF-L), glial fibrillary acidic protein (GFAP), calcium-binding protein S100B (S100B), ubiquitin carboxyterminal hydrolase L1 (UCH-L1), total tau, and cytokines (tumor necrosis factor alpha, interleukins 6 and 10)

The exploratory objectives are:

1. To explore the predictive value of the different MRI techniques (see details under the section of “Data collection and management”) alone and combined for clinical outcome at 3 months, and also at 1 and 2 years after SAH.
2. To examine the pathophysiological pathways of the post-SAH period between the two treatment groups and to assess the predictive value of selected proteomics and metabolomics on severity of EBI and DCI and neurological outcome (i.e., good outcome: mRS 0–2 vs poor outcome: 3–6)
3. To evaluate the role of microglial activation (see details under the section of “Data collection and management”) on the development of DCI.
4. To explore the role of the cytokine level on the development of DCI
5. To assess a combination of selected brain imaging markers obtained from different MRI techniques (see details under the section of “Data collection and management”), biomarkers (see secondary objectives # 8), plasma metabolomics (see details under the section of “Data collection and management”), CSF metabolomics and clinical examinations (e.g., motor score, pupillary light reflex with pupillometry, epileptic seizures) as a prognostication model for EBI, DCI, and neurological outcome at 3 months, at 1 and 2 years after SAH (i.e., good outcome: mRS 0–2 vs poor outcome: 3–6).
6. To test the applicability of utilizing a brain network analysis with graph theoretical analysis and anatomical connectivity of white matter tracts (see details under the section of “Data collection and management”) in prognostic models for EBI, DCI, and neurological outcome at 3 months, at 1 and 2 years after SAH (i.e., good outcome: mRS 0–2 vs poor outcome: 3–6) and to investigate xenon’s effect on the connectivity.
7. To unravel the mechanisms underpinning the development of EBI and DCI by utilizing brain imaging obtained from different MRI techniques (see details under the section of “Data collection and management”) and metabolomics (i.e., plasma and spinal fluid; see details under the section of “Data collection and Management”)
8. To measure the clinical neurological outcome at 3 months and at 1 year and at 2 years.
9. To develop a prognostication model for EBI and DCI and neurological outcome at 3 months and at 1 year and at 2 years after SAH

### Trial design {8}

The Xe-SAH study is a prospective, multicenter phase II drug intervention trial. The study design is a single-blind, prospective superiority randomized two-armed parallel follow-up study. The primary objective of the study is to explore the potential neuroprotective effects of xenon, when administered within 6 h of the initial insult, in adult (≥ 18 years of age), mechanically ventilated aSAH patients with a Hunt-Hess grading of 3–5. There are two study arms: the control arm receiving the current standard of care while the intervention arm will be administered a 24-h xenon treatment in addition to the standard care.

## Methods: participants, interventions, and outcomes

### Study setting {9}

Three university hospitals across Finland will participate in this study. The participating hospitals are Turku University Hospital, Tampere University Hospital, and Kuopio University Hospital. All patients with a new aSAH, admitted to the emergency room of each of the participating hospitals, will be screened for eligibility to be included in this trial.

### Eligibility criteria {10}

The inclusion criteria are:

1. Informed consent obtained from the next of kin or legal representative
2. Aneurysmal subarachnoid hemorrhage visible on CT, CTA, or DSA
3. Hunt-Hess grading of 3–5
4. Age of ≥ 18 years
5. The patient has been intubated
6. GCS 3–12 obtained off neuromuscular blocking agents
7. Xenon treatment can be initiated within 6 h after the onset of aSAH symptoms

The exclusion criteria are:

1. Acute traumatic brain injury
2. Maximum diameter of intracerebral hemorrhage > 2.5 cm
3. Pneumothorax or pneumomediastinum
4. Acute lung injury requiring ≥ 60% Fi02 (fraction of inspired oxygen)
5. Systolic arterial pressure < 80 mmHg or mean arterial pressure < 60mmHg for over a 30-min period
6. Bilaterally fixed and dilated pupils
7. Positive pregnancy test, known pregnancy, or currently breast-feeding
8. Chronic neurological deficiency due to chronic traumatic brain injury or other neurological illness
9. Imminent death or current life-threatening disease
10. Current enrollment in another interventional study
11. The subject is known to have a clinically significant laboratory abnormality, medical condition, or social circumstance that, in the investigator’s opinion, makes it inappropriate for him/her to participate in this clinical trial
12. Presence of implants or foreign bodies which are not known to be MRI safe

#### Who will take informed consent? {26a}

In this study, eligible patients are comatose and in an immediate life-threatening condition. In addition, it is likely that xenon’s putative neuroprotective effect can be achieved only if xenon inhalation has been started within 6 h after onset of SAH symptoms. Therefore, obtaining a written informed consent from next of kin or a legal representative is highly time-sensitive. An alert of an incoming SAH patient will be distributed to the study physicians (on-call anesthesiologists, intensivists, and neurosurgeons) via the phone to come to the ED to allow recruitment to proceed. The patient’s next of kin will be informed of the study either in-person or by remote communication, and written consent will be obtained either in person or electronically. A written consent will also be obtained from all those patients, who will regain full legal capacity after a sufficient neurological recovery.

#### Additional consent provisions for collection and use of participant data and biological specimens {26b}

Information that characterizes the participant’s condition prior to the initiation of experimental treatment will be obtained as soon as clinically reasonable. These include knowledge of the participant’s demographics, medical history, vital signs, and oxygen saturation. The written consent includes permission to collect blood samples, spinal fluid, and radiologic image data.

The collected information will contain quantitative and qualitative data of the patients, as stated in recent recommendations issued by the working group on subject characteristics, and will include all relevant Common Data Elements (CDE) (138). Specific definitions, measurements tools, and references regarding each SAH CDE can be found on the weblink here: https://www.commondataelements.ninds.nih.gov/sites/nindscde/files/Doc/Stroke/CDEStartupResource_Stroke.pdf.

Surgical interventions, additional detailed information related to SAH (i.e., CDE), changes in clinical status, all routinely monitored signs (i.e., arterial blood pressure, heart rate, intracerebral pressure), collected laboratory tests, and concurrent medical therapy given to the patient during the ICU stay will be recorded. GCS score, concomitant medications, surgical interventions, and neurologic conditions are collected daily during the hospitalization.

This clinical study protocol adheres to the SPIRIT reporting guidelines [45].

## Interventions

### Explanation for the choice of comparators {6b}

Despite improvements in medical technology and advances in medical science, aSAH mortality and disability rates have remained virtually the same during the past 10 years [7]. The standard of care in Finland for aSAH follows the standardized best practice guidelines set out by the American Heart Association and the American Stroke Association [46] and is the gold standard and in everyday use in the hospitals at which this trial will be conducted. The standard state-of-the-art care is therefore justified as the comparator for which this study seeks to find an improvement.

### Intervention description {11a}

After successful recruitment into the study, the patients will be randomized into one of two arms:

- The first arm is the current standard of care accompanied with normothermia (i.e., 36.5–37.5 °C).
- The second arm is the xenon intervention group, where the participants will receive inhaled xenon for 24 h with an end-tidal concentration of 50 ± 2% and normothermia (i.e., 36.5–37.5 °C) and the standard of care thereafter.

The intervention arm will receive xenon administered at 50 ± 2% in oxygen for 24 h upon admission to the intensive care unit. The concentration of xenon administered will be monitored at both the inspiratory and expiratory limbs of the rebreathing circuit using a thermoconductivity analyzer, which is automatically calibrated when the delivery system is initiated. If the concentration of xenon administered drops below 45%, the system will be flushed to displace any nitrogen emerging from blood and tissues into the closed-circuit system. Flushing will be continued until a xenon concentration of at least a 50% is reached. Xenon will be administered for 24 h through the rebreathing circuit. If the patient should require surgery for any reason during the xenon inhalation treatment, the procedure will be performed under xenon anesthesia whenever feasible. Supplemental sedative, anesthetic, and pain medication are to be provided as necessary during surgery. A failure to maintain a 50% concentration of xenon in oxygen during the treatment phase is not a reason to disqualify the patient from the study. In such a case, the treatment will be continued with the safest concentration that can be administered. The end-tidal xenon concentration of 70% may not be exceeded nor can it be less than 30% (i.e., the upper safety limit and lower efficacy limit of xenon concentration).

Since acute hydrocephalus is relatively common after aSAH, most of the recruited patients will receive an external ventricular drain. ICP monitoring will also be performed in all patients. ICP target is set to ≤ 20 mmHg; an optimal head position of 30° will be ensured. The ICP monitoring device must be in place prior to the commencement of xenon inhalation. Mechanical ventilation and adjustments will be made based on regular treatment. The inspiratory oxygen will be adjusted to maintain a partial pressure for arterial oxygen in the range of 12–20 kPa. Minute ventilation will be adjusted to maintain the partial pressure for arterial carbon dioxide in the range of 4.5–5.5. kPa. Repeated blood-gas analysis will be performed every hour during the xenon treatment, and later whenever indicated but at least every 4 h. The following hemodynamic targets are set: cerebral perfusion pressure (CPP) > 60mmHg, mean arterial pressure (MAP) > 65mmHg. Management of hemodynamics will be primarily achieved through the use of vasopressors (e.g., norepinephrine, epinephrine, dopamine, dobutamine), and antiarrhythmic medications will be administered as needed and according to local protocols. Hypertension will be treated with vasodilators.

Serum glucose-targets are set to between 5.0 and 10.0 mmol/L and hematocrit between 0.3 and 0.45. Potassium will be supplemented if the serum potassium level is < 4 mmol/L. Plasma sodium levels will be maintained above 135 mmol/l and will not be corrected if osmotic therapy for intracranial hypertension results in an elevation of plasma sodium, unless it exceeds 155 mmol/l. Normothermia of 36.5–37.5 °C will be maintained after ICU arrival. The core temperature will be managed with infusions of diclofenac and paracetamol according to clinical routine at each center.

### Criteria for discontinuing or modifying allocated interventions {11b}

The study of an individual patient will be terminated by investigator or by the attending physician at any moment during the treatment in the ICU if the safety of the patient cannot be otherwise assured. Thereafter, the patient will be treated by the most appropriate means according to the judgment of the attending physician. Each individual case will be considered by the attending physician according to clinical evidence of intensive care medicine.

The study subjects may be withdrawn from their study treatment prematurely by the investigator or by the physician for one or more of the following reasons:

- A failure to maintain a xenon concentration ≥ 30 %
- A failure of ventilation and/or oxygenation of the patient with the xenon delivery device
- Adverse event (AE)/serious adverse event (SAE)
- Protocol violation
- If for any reason the investigator or the attending physician believes that continued participation in the study is not in the best interests of the patient.

### Strategies to improve adherence to interventions {11c}

None. Due to the nature of the study, the 24-h xenon treatment will be given to the intervention group and MR imaging will be conducted on those patients where the imaging can be safely performed.

### Relevant concomitant care permitted or prohibited during the trial {11d}

Standard of care, no other special provisions.

### Provisions for post-trial care {30}

Standard of care for all. The treatment studied and research-related procedures are free of charge for study participant. Any loss of earnings and travel expenses caused by the examination visits will be reimbursed to a patient/family member/relative according to the actual costs based on supporting documents. If the study subject suffers personal injury as a result of the study treatment or an action taken as a result of the study, one can apply for compensation. Further details are given in the informational documentation handed out to both the patient and next of kin.

## Outcomes {12}

### Primary outcome measure

1. Global fractional anisotropy of white matter of diffusion tensor imaging (DTI). White matter damage will be less severe in xenon-treated patients, i.e., global fractional anisotropy will be significantly higher in the xenon group than in the control group as assessed with the 1st MRI.

### Secondary outcome measures

1. Fractional anisotropy of white matter at cerebellum and/or at corpus callosum as assessed with the 1st MRI.
2. Safety and tolerability of xenon, as assessed with a ratio of adverse events, serious adverse events, and suspected unexpected serious adverse reactions (SUSARSs) during the follow-up at 1 year between the xenon group and the control group.
3. Composite of radiological EBI (within 72 h after the start of SAH symptoms), DCI, cerebral infarction (see definitions in detail under the section of secondary objectives) and poor outcome (good: mRS 0–2; poor: mRS 3–6) at 3-months and at 1 year.
4. Neurogenic stress cardiomyopathy and stunned myocardium (i.e., myocardial injury caused by sympathetic storm and autonomic dysregulation with hs-troponin elevation, left ventricular dysfunction or ECG changes)
5. ICP-level up to 14 days (i.e., as long as clinically relevant) after the onset of aSAH symptoms
6. Need for ICP therapies up to 14 days (i.e., as long as clinically relevant) after the onset of aSAH symptoms
7. Duration of therapy for ICP control/monitoring up to 14 days (i.e., as long as clinically relevant) after the onset of aSAH symptoms
8. Plasma catecholamine levels
9. Difference in selected biomarkers (NSE, NF-L, GFAB, S100B, UCH-L1, total-tau TNF-α, interleukins 6 and 10) and difference in metabolomic and lipidomic profiles in the blood and CSF between xenon and control group in predicting risk for EBI, vasospasm, DCI as well as a good neurological outcome at 3 months, 1 year, and at 2 years after the aSAH.
10. Development of prognostication models with a selected combination of brain imaging and biomarkers obtained from various MRI techniques (see details under the section “Data collection and management”), clinical data, and metabolomics (see details under the section “Data collection and management”) by applying artificial intelligence and machine learning for EBI, vasospasm, DCI, and a good neurological outcome at 3 months, 1 year, and at 2 years after the aSAH.
11. Difference in CTA, DSA, and MRI parameters obtained from various MRI techniques (see details under the section “Data collection and management”) between xenon and control group in predicting EBI, vasospasm, DCI, and a good neurological outcome at 3 months, 1 year, and at 2 years after the aSAH.
12. Predictive value of computational fluid dynamics (CFD) (see details under the section “Data collection and management”) to detect the differences in the blood flow behavior in the recruited patients by applying 3D DSA to assess the risk of EBI within the first 72 h after the start of aSAH symptoms, vasospasm (within 21 days after the start of SAH symptoms) and DCI (between day 4 and 6 weeks after the start of SAH symptoms) and a good neurological outcome at 3 months, at 1 year, and at 2 years after the aSAH (mRS 0–2).
13. Difference of metabolomics and lipidomics profiles of plasma and CSF between xenon and control group and in predicting the risk for EBI within the first 72 h after the start of aSAH symptoms, vasospasm (within 21 days after the start of SAH symptoms) and DCI (between day 4 and 6 weeks after the start of SAH symptoms) and a good neurological outcome at 3 months, at 1 year, and at 2 years after the aSAH (mRS 0-2).
14. Difference of activity of microglial cells between the subjects in the xenon and control groups and in predicting the risk for DCI and a good neurological outcome at 3 months, 1 year, and at 2 years after the aSAH.

## Participant timeline {13}

**Fig. 1:**
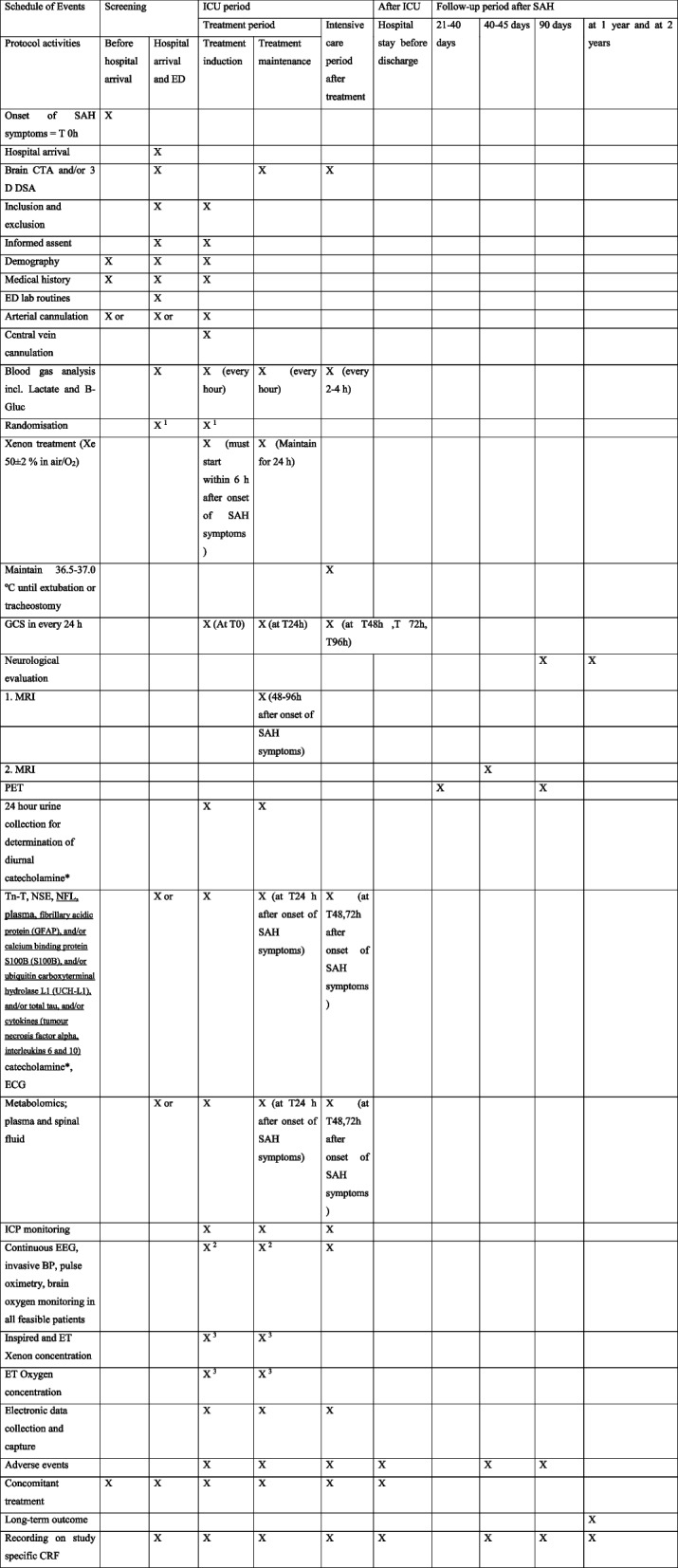
Participant timeline

The schedule of events for study participants, including enrolment, interventions, and assessments, is outlined in Fig. 1.

## Sample size {14}

A sample size of 100 is estimated on the basis of recent studies in aSAH patients as being able to provide 80% power with a 2-sided α level of 0.05 in order to detect a mean difference of 0.02 (SD 0.035) in the global fractional anisotropy of white matter between the xenon-treated group and the control group [44]. In addition, this mean difference is estimated to have a predictive value for DCI and poor neurological outcome. It is estimated that approximately 30% of the enrolled patients will be lost from the MRI studies, and therefore, 80 patients per group will be enrolled in this study.

## Recruitment {15}

A team of anesthesiologists and neurosurgeons at each study site will be responsible for setting up and organizing the swift identification of potential SAH patients that arrive in the emergency department and they will have a contingency plan in place to ensure equally rapid screening for eligibility, obtaining of written consent and recruitment.

The primary recruitment center for the start of this study will be Turku University Hospital; the other centers are expected to join after the feasibility of the protocol has been assured. The expected rate of recruitment is 20 patients, consisting of both the Xenon-intervention arm and standard-of-care arm, per year at Turku University Hospital. Tampere University Hospitals expected rate of recruitment is comparable to Turku, with 20 patients per year. Kuopio University hospital’s rate of recruitment is expected to be 10 patients per year. Thus, the expected recruitment phase of the study is expected to last 4 years.

## Assignment of interventions: allocation

### Sequence generation {16a}

After screening, an eligible patient will be included in the study and randomly allocated into either the standard of care arm or the xenon intervention arm in a ratio of 1:1. Informed written consent of participation will be received from the next of kin or the legal representative of the included patient as soon as possible. Trial sites will have access to an Internet-based randomization system. Each subject will be assigned a unique study and randomization number. A block randomization approach will be stratified by the study site.

### Concealment mechanism {16b}

Randomization will be Internet-based, and the use of a validated password website will ensure concealment.

### Implementation {16c}

The Xe-SAH -trial comprises the following scheduled events: screening, ICU stay (treatment induction and maintenance and post-treatment), hospital stay after ICU stay, and follow-up at 3, 12, and 24 months after the SAH. In order to confirm the feasibility of the protocol (i.e., period of feasibility), the trial will be initiated in the intensive care unit of Turku University Hospital in close liaison with the Departments of Neurosurgery and Neuroradiology. We expect that the period of feasibility will be completed within 3–6 months. Thereafter, the trial will also be started in Tampere and Kuopio University Hospitals. Implementation will be the responsibility of the senior physicians and researchers from the Departments of Anesthesiology and Intensive Care, Neurosurgery, and Neuroradiology at each participating center.

## Assignment of interventions: blinding

### Who will be blinded? {17a}

The physicians responsible for assessing the neurologic outcome as well as the neuroradiologist analyzing the brain imaging will be unaware of the treatment-arm. The immediate care-providers, including the attending physician and their team, cannot be blinded during the intervention phase, due to logistical and treatment effects related to xenon-administration.

### Procedure for unblinding if needed {17b}

The need for unblinding should not arise, as the immediate care-providers are not blinded. Only the neuroradiologist and neurologist who assess the primary and/or secondary outcomes are blinded.

## Data collection and management

### Plans for assessment and collection of outcomes {18a}

Upon hospital arrival and thereafter, basic patient information will be recorded in an electronic web-based study database (RedCap). The data collected on aSAH patients is intended to be both quantitative and qualitative in nature, as recommended by the recent recommendations issued by the working group on subjects’ characteristics, including all relevant Common Data Elements (CDE) [47, 48]. The data to be collected will include details of the patient’s age, sex, home medications and health status, time of insult, hospital arrival delay, vital signs, and blood tests. First computed tomography (CT), computed tomography angiography (CTA), and digital subtraction angiography (DSA) results will be registered in the web-based study’s database according to CDE specifications. During the ICU stay, surgical interventions, vital signs, changes in clinical status, and other medical treatments received will be recorded. The duration of xenon administration will be recorded. In addition, arterial blood gas samples will be monitored hourly during the xenon-treatment phase, and then at least every 4 h or whenever clinically indicated during the ICU stay. The results of the MRI scans at 72 ± 24 h and 42 ± 4 days after the onset of SAH symptoms will be collected in detail according to the CDE recommendations.

The following MRI techniques will be used: (1) T2-weighted and fluid-attenuated inversion recovery (FLAIR) sequences; (2) T1-weighted 3D gradient echo sequence for volumetric analysis; (3) time of flight angiography (TOF) to visualize flow within vessels, without the need to administer a contrast agent; (4) brain perfusion imaging with dynamic susceptibility contrast (DSC), (5) a susceptibility-weighted imaging (SWI) sequence to image hemosiderin, (6) whole-brain (and region-of-interest level) white matter integrity from diffusion-weighted MRI data measured with diffusion tensor imaging (DTI; fractional anisotropy is the primary end-point of the current trial; (7) voxel-wise white matter microstructure from diffusion-weighted MRI data. A global voxel-wise data-driven Tract Based Spatial Statistics (TBSS) with DTI and other microstructural metrics will be applied; (8) constrained spherical deconvolution (CSD) technique will be used to estimate the orientation of multiple fiber populations, e.g., crossing fibers, in a voxel accurately and within a clinically feasible scan time. The fiber orientation distributions will be estimated with CSD and used to perform probabilistic fiber tractography for the whole brain and for certain tracts of interest. The whole brain tract networks will be analyzed with graph theoretical analysis [49–53].

In addition, all feasible patients (i.e., capable to co-operate adequately during the scan) will be scanned in the Turku PET Centre. The 1st scan will be performed 4 ±1 weeks after the onset of SAH symptoms with the 2nd scan being undertaken at 3 months after the onset of SAH symptoms. It will be explored whether [^11^C](R)-PK11195 can be used to test the hypothesis that xenon can exert a neuroprotective effect and to explore the role of inflammatory process in the extent of DCI and also the neurological outcome at 1 year after SAH. We postulate that this can be demonstrated by showing less signs of microglial activation in the xenon-treated group than in the reference therapy group and in the patients with good outcome, i.e., mRS 0–2 [54–56].

Metabolomics will be applied to identify pathophysiological pathways after SAH governing survival, to identify differences in targeted metabolomic pathways between the two treatment groups and discover new biological biomarkers or specific pathways for prognostic purposes. We intend to perform both (i) comprehensive metabolomics analyses using two methods with a broad analytical coverage, for molecular lipids (lipidomics) and polar metabolites, respectively, and (ii) selected targeted/quantitative methods for functionally important metabolites of CNS origin. Among the latter, the proposed methods will include N-acyl amides (NAAs, a class of molecules that encompasses the endocannabinoids and related molecules) and ceramides [57–61].

Human blood vessels will be investigated using computational fluid dynamics (CFD) to detect the differences in blood flow behavior in the recruited patients. CFD will be applied in the dynamic 3D DSA imaging data of the current population with the aim being to investigate and create models of blood flow behavior in the proximity of the ruptured aneurysms, and in distant regions as well as globally. Artificial intelligence (AI) and machine learning (ML) will be used by combining the results of CFD simulations and clinical data to create models which possess a high predictive accuracy for estimating the risk for EBI, vasospasm, and DCI and poor neurological outcome in aSAH patients. The predictive accuracy of the prognostication models created in derivation cohorts of the retrospective population will be tested in retrospective validation cohorts and in individual cases, and in the prospective Xe-SAH population. The retrospective population (initially collected by JK and IS) will comprise all consecutive aneurysm cases which will have been detected in the catchment area of the Hospital District of Southwest Finland from January 2000 to May 2018 (i.e., Turku Aneurysm Data, TAD). Altogether, over 2600 unique aneurysm patients have been recorded into TAD. In all cases, the automatic text mining will be performed by Auria Biobank Service (Turku, Finland) registering all the clinical, radiological, laboratory, and other available events throughout the history of each individual patient before and after the diagnosis of the aneurysm. The total amount of events in the TAD constitutes over 70,000,000.

### Plans to promote participant retention and complete follow-up {18b}

Only routine encouragement if necessary.

### Data management {19}

Data collected upon arrival and all neuroradiology findings will be stored in an electronic web-based study database (Redcap, https://www.project-redcap.org/ [62, 63]). This data will be securely stored at the servers of the corresponding city’s universities where the patients are being treated. Intensive care data, including blood pressure, blood samples, and ICP data for example will be stored at a secure server at the corresponding University Hospitals in accordance with hospital guidelines and the Data Management Plan as described below in this protocol.

Access to clinical data with patient IDs is strictly limited to the clinicians treating the patients and maintaining the database. Exports of the clinical data for analyses are always pseudononymized to protect patient confidentiality. The imaging data exports are stored only at MRI unit and Turku PET Centre of Turku University Hospital, which are audited workspace environments that is designed to host sensitive data for collaboration. All researchers sign a non-confidentiality agreement with strict rules on data handling before they are granted access to the exports. The study sites have ethical review board approvals for collection and working with clinical material and data.

All data with direct or indirect identification information will be stored in encrypted form and a separately stored decryption code will be stored with access by the PI. Essential documents shall be archived safely and securely.

Each study patient is identified by a patient code (i.e., first two initials of first and last name and subject number). The patient identity (initials and screening/subject number, the subject’s first name, last name, date of birth, social security number) is recorded on a separate register (i.e., patient identity file) which can be accessed only by the PI with a password. Study subject records, subject screening log, all original signed informed consent forms, and CRFs will be kept in the Investigator’s study file to enable the follow-up assessments by safety committee and monitor. Investigator’s study file can be accessed only by the PI and biostatistician with a password.

The study subject code (initials and screening/subject number), date of informed consent, date of entry or date of exclusion, and the reason for exclusion for those recruited for screening but not fulfilling the inclusion criteria and therefore excluded will be recorded in the subject screening log.

### Confidentiality {27}

Data is stored in accordance with local data protection guidelines applying the data management plan as described in the protocol; see the section “Data collection and management”.

### Plans for collection, laboratory evaluation, and storage of biological specimens for genetic or molecular analysis in this trial/future use {33}

Biochemical assessment: A blood sample of 20 ml for determination of plasma catecholamines, plasma metabolomics (see details of metabolomics under the section “Data collection and management”), cardiac enzyme release (P-hs-troponin-T and heart fatty-acid binding protein), neuron-specific enolase (NSE), neurofilament light (NF-L), glial fibrillary acidic protein (GFAP), calcium-binding protein S100B (S100B), ubiquitin carboxyterminal hydrolase L1 (UCH-L1), total tau, and cytokines (tumor necrosis factor alpha, interleukins 6 and 10) will be analyzed at ICU arrival and at 24h, at 48h, and at 72h after onset of SAH symptoms; urine catecholamines during first and second 24 h. In addition, a sample of spinal fluid will be collected through EVD at ICU arrival or as soon as it is in place and at 24h, at 48h, and at 72h after onset of SAH symptoms for assessment of metabolomics. P-hsTn-T and P-NSE will be determined by a local university hospital laboratory with standard methods. The samples will be destroyed 10 years after completion of the study. In addition, the patient has a right to require destruction of the samples at any time. In this study, no tissue samples will be collected, and no genetic analysis are conducted.

## Statistical methods

### Statistical methods for primary and secondary outcomes {20a}

The primary outcome measure is a global fractional anisotropy of white matter of diffusion tensor imaging (DTI) as assessed in the 1st MRI. The univariate distributions of variables and the associations between variables will be described using summary statistics. Basic statistical tests (two-sample *t*-test, Mann-Whitney *U*-test, chi-square test, and Fisher exact test) will be used in the preliminary analysis to compare the xenon-treated and control groups. Kaplan-Meier curves and Cox regression analyses will be applied when examining the occurrence of end-point events. An analysis of variance of repeated measurements will be applied for normally distributed outcome variables. In the case of other types of variables, the analysis will be based on generalized linear mixed models which include comprehensive and general tools to analyze longitudinal data. General linear model for normally distributed outcomes and generalized linear models for other types of variables will be used for analyzing data at different time points. The analyses will be adjusted for age, sex, and site, and other baseline characteristics of patients will be used as covariates when needed. The adjusted analysis will be considered to be the main analysis. The statistical analysis will be based on all available data. If an analysis is based only on complete cases, it will be mentioned separately. The data will be analyzed according to intention-to-treat and per-protocol populations. The differences will be presented as mean differences with 95% confidence intervals for continuous outcomes, odds ratios with 95% confidence intervals for categorical outcomes, and hazard ratios with 95% confidence intervals in survival analyses. A two-sided statistical test with a significance level of 0.05 will be used in the statistical analyses.

### Interim analyses {21b}

No interim analysis will be performed. However, an interim analysis can be performed if requested by the safety board, e.g., should early termination of the trial be considered due to a suspicion of compromised patient safety by xenon or by other reasons.

### Methods for additional analyses (e.g., subgroup analyses) {20b}

A sub-study will be performed in patients with a delay to randomization of no more than 3 h after the start of aSAH symptoms.

### Methods in analysis to handle protocol non-adherence and any statistical methods to handle missing data {20c}

The data will be analyzed according to intention-to-treat analysis. The extent of possible missing data will be analyzed and examined, especially considering the primary outcome of the study.

### Plans to give access to the full protocol, participant-level data, and statistical code {31c}

The data of this study will be available to investigators whose proposed use of the data has been approved by an independent review committee. Individual participant data that underlie the results reported in this Article will be shared (text, tables, figures, and appendices), after de-identification, along with the study protocol. These data will be available 6 months after the article’s publication and will be available for 12 months from publication. Data can be used for individual participant data meta-analysis. Requests and proposals should be directed to timo.laitio@tyks.fi. To gain access, data requestors will need to sign a data access agreement.

## Oversight and monitoring

### Composition of the coordinating center and trial steering committee {5d}

Turku University Hospital will be the coordinating center. The trial steering committee will be chaired by the principal investigator Timo Laitio and will comprise site principal investigators and one head physician or investigator from the Departments of Anesthesia and Intensive Care, Neurosurgery, Neuroradiology, Cardiology, and Turku PET Centre.

There is no Stakeholder or Public Involvement Group associated with this trial.

### Composition of data monitoring committee, its role and reporting structure {21a}

Data and Safety Monitoring Plan will be prepared and a Data and Safety Monitoring Board (i.e., independent safety committee) will be established according to the relevant Standard Operational Procedures of Turku Clinical Research Counsel; the plan and the composition of the board will be communicated with the National Committee on Medical Research Ethics (TUKIJA) before the commencement of this study.

In addition, TUKIJA will be informed about outcomes related to the responsibilities of the Data and Safety Monitoring Board and about other responsibilities defined by ICH good clinical practice (GCP).

The study monitor will visit the study center after enrollment of every 4 patients and after each 6-month interval. The study monitor will ensure that the study is complying with the GCP and applicable regulatory requirements and that the protocol is being adhered to in all aspects, with accurate recording of results, reporting of AEs, drug accountability, and record keeping.

Furthermore, it will be verified that the clinical facilities remain adequate and that the CRFs correspond with source data. For this purpose, the study monitor will be allowed direct access to hospital or patient records of the study subject, original laboratory data, etc., relevant to the study. It is essential that the investigator and other relevant members of the study center team are available during the monitoring visits and inspections, and that they can devote sufficient time to these processes.

### Adverse event reporting and harms {22}

Study subjects participating in this study are already in need of intensive care due to one or more signs of organ insufficiency or failure. Therefore, multiple and diverse clinical symptoms and laboratory findings must be anticipated to occur frequently. Expected minor fluctuations in the study patients’ presenting illness will not represent an AE. Any clinically significant worsening in a study patient’s condition according to clinical judgment, laboratory finding, or other diagnostic findings, compared with the study patient’s baseline status at the time of starting xenon treatment will have to be recorded as an AE whether or not the worsening condition is considered to be due to the study patient’s underlying illness or study treatment. All AEs must be elicited, documented, and reported to the investigator from the moment of treatment until the end of the follow-up period of six months.

### Frequency and plans for auditing trial conduct {23}

The study monitor will visit the study center after enrollment of every 4 patients and after each 6-month interval.

### Plans for communicating important protocol amendments to relevant parties (e.g., trial participants, ethical committees) {25}

All substantial protocol amendments will be detailed in writing and consequently sent to TUKIJA (National Committee on Medical Research Ethics) and FIMEA (Finnish Medicines Agency) via the EU C-TIS Portal. If other administrative changes need to be made to the protocol, the study will be discontinued until it receives subsequent approval by TUKIJA and FIMEA.

## Dissemination plans {31a}

The findings of this study will be presented in peer-reviewed journal publications as well as conference presentations.

## Discussion

In this Xe-SAH protocol, we describe how we intend to conduct a prospective, multicenter phase II clinical drug trial. The study design is a single-blind, randomized two-armed parallel follow-up study. The primary objective of the study is to explore the potential neuroprotective effects of xenon, when administered within 6 h after the onset of symptoms of aSAH. Due to the devastating nature of an aSAH, there is an urgent need for new interventions that target EBI to potentially attenuate the secondary injury from DCI and thereby improve the neurological and the overall clinical outcome [9–12, 15, 16].

This study has multifold theoretical and clinical premises to assess the extent of neuroprotection. Firstly, at a pathophysiological level, it has been demonstrated that xenon targets multiple processes implicated in the development of brain injury after aSAH, such as glutamate-mediated excitotoxicity, neuro-apoptosis, ischemia-reperfusion injury, and oxidant injury [9, 12, 16, 30, 64–69]. Secondly, multiple experimental animal models have demonstrated xenon’s neuroprotective effect on global ischemia, focal occlusive stroke, TBI, and SAH with functional and histopathological long-term benefits on the outcome [20–24, 26–37]. Furthermore, Laitio et al. showed that xenon was able to decrease the global and regional cerebral metabolic rate of glucose (MRGlu) using positron emission tomography (PET) in healthy human volunteers [70, 71]. Additionally, the Xe-Hypotheca trial was a proof-of-concept study and the first to demonstrate xenon’s ability to attenuate the extent of a white matter injury after global ischemia, mainly due to a protective effect on myelin [43]. A white matter injury is common after an aSAH during the first 72 h after ictus and has an independent predictive value for DCI and the neurological outcome, this explains why the results of the Xe-Hypotheca trial are particularly relevant [44, 72–75]. Good white matter function is fundamental for normal neurocognitive performance. Since as many as 75 % of survivors will experience psychosocial, neurocognitive, or other neurological problems, there is a clear target population of individuals who could benefit from xenon’s putative neuroprotective effect. It is also important to note that the white matter injury after SAH has been observed with fractional anisotropy, a technique that is known to reflect axonal injury and demyelination [44, 72–80]. For that reason, the fractional anisotropy will be the primary endpoint of this study.

As a secondary endpoint of the Xe-HypotheCA trial, inhaled xenon combined with mild therapeutic hypothermia resulted in a reduced myocardial injury when compared to that achieved by hypothermia alone as demonstrated by the significantly lower release of troponin-T from baseline to 72 hours after OHCA in the xenon group. The incremental change of troponin-T was also associated with higher mortality at six months [40]. In addition, the Xe-Hypotheca trial revealed that inotropic support with norepinephrine during the first 72 h after cardiac arrest was 84.3% higher in the control group than in the patients treated with xenon. Among aSAH patients, 35% displayed elevations of troponin I, 35% experienced arrhythmias, and 25% exhibited ventricular wall motion abnormalities with a catecholamine-induced process proposed to be the most plausible mechanism behind the cardiac complications [5, 81–84]. It is known that the cardiac manifestations, which usually persist for 1–3 days, are more common in patients who will later develop DCI, and these are associated with worse outcomes. A clinical syndrome termed Neurogenic Stress Cardiomyopathy (“stunned myocardium”) may contribute to death in 12% of aSAH patients. It is also well-documented that high levels of catecholamines associate with cardiac dysfunction, late vasospasm, and poorer outcome [85–91]. Therefore, xenon’s cardioprotective and inotrope-sparing effects may prove to be beneficial after an aSAH.

The start of the trial has been postponed due to the respirator shortage caused by the corona pandemic, but it is now expected to start during 2023. Patient recruitment will start initially (i.e., a feasibility period of 3–6 months) in Turku University Hospital, with Tampere and Kuopio University Hospitals joining in at a later stage after the feasibility period. Patient recruitment is expected to last four (4) years and the neurological follow-up surveys will require an additional two (2) years. A feasibility study will be published, once 30 patients have successfully gone through the randomization process and received treatment. One of the main endpoints of the feasibility study is to demonstrate the safety of xenon when assessing ICP.

Due to the devastating nature of an aSAH, there is an urgent need for new neuroprotective strategies to reduce both the mortality and morbidity that are associated with aSAH. In this respect, this study aims to deliver new interventional strategies for the treatment of aSAH.

## Trial status

Recruitment for the trial is expected to begin in Turku University Hospital on the 1st of October 2023. The two other research sites in Finland, Tampere and Kuopio University Hospitals, are expected to join once the feasibility of the protocol has been assured. The estimated date for finishing the recruitment phase is October 2027.

## Data Availability

The data of this study will be available to investigators whose proposed use of the data has been approved by an independent review committee. The individual participant data that underlie the results can be shared (text, tables, figures, and appendices), after de-identification, along with the study protocol and after publication. These data will be available at 6 months after the publication and will be available for 12 months from publication. Data can be used for individual participant data meta-analysis. Requests and proposals should be directed to timo.laitio@elisanet.fi. In order to gain access, data requestors will need to sign a data access agreement.

## Abbreviations

AE: Adverse events
CBF: Cerebral blood flow
CDE: Clinical data element
CPP: Cerebral perfusion pressure
CT: Computer tomography
CTA: Computer tomography angiography
DCI: Delayed cerebral ischemia
DSA: Digital subtraction angiography
DTI: Diffusion tensor imaging
EBI: Early brain injury
EVD: External ventricular drainage
FI02: Fraction of inspired oxygen
FA: Fractional anisotropy
ICP: Intracranial pressure
ICU: Intensive care unit
MAC: Minimal alveolar concentration
MR: Magnetic resonance
MRA: Magnetic resonance angiography
NMDA: N-Methyl-D-aspartate
NFL: Neurofilament light chain
NSE: Neuron-specific enolase
PEEP: Positive end-expiratory pressure
PET: Positron emission tomography
SAE: Serious adverse event
SAH: Subarachnoid hemorrhage
aSAH: Aneurysmal subarachnoid hemorrhage
SUSAR: Suspected unexpected serious adverse reaction
TnT: Troponin-T
TOF: Time-of-flight
Xe: Xenon

## References

[1] Lawton MT, Vates GE (2017). Subarachnoid hemorrhage. N Engl J Med..

[2] van Gijn J, Kerr RS, Rinkel GJ (2007). Subarachnoid haemorrhage. Lancet..

[3] Connolly ES, Rabinstein AA, Carhuapoma JR, Derdeyn CP, Dion J, Higashida RT (2012). Guidelines for the management of aneurysmal subarachnoid hemorrhage: a guideline for healthcare professionals from the american heart association/american stroke association. Stroke..

[4] Suarez JI (2015). Diagnosis and management of subarachnoid hemorrhage. Contin Lifelong Learn Neurol..

[5] Diringer MN, Bleck TP, Hemphill JC, Menon D, Shutter L, Vespa P (2011). Critical care management of patients following aneurysmal subarachnoid hemorrhage: Recommendations from the neurocritical care society’s multidisciplinary consensus conference. Neurocrit Care..

[6] Udy AA, Vladic C, Saxby ER, Cohen J, Delaney A, Flower O (2017). Subarachnoid hemorrhage patients admitted to intensive care in Australia and New Zealand: a multicenter cohort analysis of in-hospital mortality over 15 years. Crit Care Med..

[7] Sharma D (2020). Perioperative management of aneurysmal subarachnoid hemorrhage: a narrative review. Anesthesiology..

[8] Korja M, Silventoinen K, Laatikainen T, Jousilahti P, Salomaa V, Hernesniemi J (2013). Risk factors and their combined effects on the incidence rate of subarachnoid hemorrhage - a population-based cohort study. PLoS One.

[9] Fujii, M., Yan, J., Rolland, W. B., Soejima, Y., Caner, B., & Zhang, J. H. (2013). Early brain injury, an evolving frontier in subarachnoid hemorrhage research. In Translational Stroke Research (Vol. 4, Issue 4, pp. 432–446). Springer US. 10.1007/s12975-013-0257-2. 10.1007/s12975-013-0257-2 PMC3719879 23894255

[10] Savarraj J, Parsha K, Hergenroeder G, Ahn S, Chang TR, Kim DH (2018). Early brain injury associated with systemic inflammation after subarachnoid hemorrhage. Neurocrit Care..

[11] Cahill WJ, Calvert JH, Zhang JH (2006). Mechanisms of early brain injury after subarachnoid hemorrhage. J Cereb Blood Flow Metab..

[12] Geraghty JR, Testai FD (2017). Delayed cerebral ischemia after subarachnoid hemorrhage: beyond vasospasm and towards a multifactorial pathophysiology. Curr Atheroscler Rep.

[13] Dorhout Mees, S. M., Rinkel, G. J. E., Feigin, V. L., Algra, A., van den Bergh, W. M., Vermeulen, M., & van Gijn, J. (2007). Calcium antagonists for aneurysmal subarachnoid haemorrhage. In Cochrane Database of Systematic Reviews (Issue 3). 10.1002/14651858.CD000277.pub3. 10.1002/14651858.CD000277.pub3 PMC7044719 17636626

[14] Dorhout Mees, S. M., van den Bergh, W. M., Algra, A., & Rinkel, G. J. E. (2007). Antiplatelet therapy for aneurysmal subarachnoid haemorrhage. In Cochrane Database of Systematic Reviews (Issue 2). 10.1002/14651858.CD006184.pub2. 10.1002/14651858.CD006184.pub2 PMC8919458 17943892

[15] Rowland MJ, Hadjipavlou G, Kelly M, Westbrook J, Pattinson KTS (2012). Delayed cerebral ischaemia after subarachnoid haemorrhage: Looking beyond vasospasm. Br J Anaesth.

[16] Foreman B (2016). The pathophysiology of delayed cerebral ischemia. J Clin Neurophysiol.

[17] Vergouwen MDI, Vermeulen M, van Gijn J, Rinkel GJE, Wijdicks EF, Muizelaar JP (2010). Definition of delayed cerebral ischemia after aneurysmal subarachnoid hemorrhage as an outcome event in clinical trials and observational studies: proposal of a multidisciplinary research group. Stroke.

[18] Goto T, Suwa K, Uezono S, Ichinose F, Uchiyama M, Morita S (1998). The blood-gas partition coefficient of xenon may be lower than generally accepted. Br J Anaesth.

[19] Sanders RD, Franks NP, Maze M (2003). Xenon: No stranger to anaesthesia. Br J Anaesth.

[20] Veldeman M, Coburn M, Rossaint R, Clusmann H, Nolte K, Kremer B (2017). Xenon reduces neuronal hippocampal damage and alters the pattern of microglial activation after experimental subarachnoid hemorrhage: a randomized controlled animal trial. Front Neurol.

[21] Franks N, Dickinson R, de Sousa S, et al. How does xenon produce anaesthesia?. Nature. 1998;396:324. 10.1038/24525. 10.1038/24525 9845069

[22] Wilhelm S, Ma D, Maze M, Franks NP (2002). Effects of xenon on in vitro and in vivo models of neuronal injury. Anesthesiology.

[23] Ma D, Wilhelm S, Maze M, Franks NP (2002). Neuroprotective and neurotoxic properties of the “inert” gas, xenon. Br J Anaesth.

[24] Ma D, Yang H, Lynch J, Franks NP, Maze M, Grocott HP (2003). Xenon attenuates cardiopulmonary bypass-induced neurologic and neurocognitive dysfunction in the rat. Anesthesiology..

[25] Ma D, Hossain M, Rajakumaraswamy N, Franks NP, Maze M (2003). Combination of xenon and isoflurane produces a synergistic protective effect against oxygen-glucose deprivation injury in a neuronal-glial co-culture model. Anesthesiology.

[26] Homi HM, Yokoo N, Ma D, Warner DS, Franks NP, Maze M (2003). The neuroprotective effect of xenon administration during transient middle cerebral artery occlusion in mice. Anesthesiology..

[27] Schmidt M, Marx T, Glöggl E, Reinelt H, Schirmer U (2005). Xenon attenuates cerebral damage after ischemia in pigs. Anesthesiology..

[28] Fries M, Nolte KW, Coburn M, Rex S, Timper A, Kottmann K (2008). Xenon reduces neurohistopathological damage and improves the early neurological deficit after cardiac arrest in pigs. Crit Care Med..

[29] Dingley J, Tooley J, Porter H, Thoresen M (2006). Xenon provides short-term neuroprotection in neonatal rats when administered after hypoxia-ischemia. Stroke..

[30] Ma D, Hossain M, Chow A, Arshad M, Battson RM, Sanders RD (2005). Xenon and hypothermia combine to provide neuroprotection from neonatal asphyxia. Ann Neurol..

[31] Coburn M, Maze M, Franks NP (2008). The neuroprotective effects of xenon and helium in an in vitro model of traumatic brain injury. Crit Care Med..

[32] Campos-Pires R, Armstrong SP, Sebastiani A, Luh C, Gruss M, Radyushkin K (2015). Xenon improves neurologic outcome and reduces secondary injury following trauma in an in vivo model of traumatic brain injury. Crit Care Med..

[33] Campos-Pires R, Hirnet T, Valeo F, Ong BE, Radyushkin K, Aldhoun J (2019). Xenon improves long-term cognitive function, reduces neuronal loss and chronic neuroinflammation, and improves survival after traumatic brain injury in mice. Br J Anaesth.

[34] Fries M, Brücken A, Çizen A, Westerkamp M, Löwer C, Deike-Glindemann J (2012). Combining xenon and mild therapeutic hypothermia preserves neurological function after prolonged cardiac arrest in pigs. Crit Care Med..

[35] Chakkarapani E, Dingley J, Liu X, Hoque N, Aquilina K, Porter H (2010). Xenon enhances hypothermic neuroprotection in asphyxiated newborn pigs. Ann Neurol..

[36] Sheng SP, Lei B, James ML, Lascola CD, Venkatraman TN, Jung JY (2012). Xenon neuroprotection in experimental stroke: interactions with hypothermia and intracerebral hemorrhage. Anesthesiology.

[37] Hobbs C, Thoresen M, Tucker A, Aquilina K, Chakkarapani E, Dingley J (2008). Xenon and hypothermia combine additively, offering long-term functional and histopathologic neuroprotection after neonatal hypoxia/ischemia. Stroke.

[38] Coburn M, Kunitz O, Baumert JH, Hecker K, Haaf S, Zühlsdorff A (2005). Randomized controlled trial of the haemodynamic and recovery effects of xenon or propofol anaesthesia. Br J Anaesth.

[39] Rossaint R, Reyle-Hahn M, Schulte Am Esch J, Scholz J, Scherpereel P, Vallet B (2003). Multicenter randomized comparison of the efficacy and safety of xenon and isoflurane in patients undergoing elective surgery. Anesthesiology.

[40] Arola O, Saraste A, Laitio R, Airaksinen J, Hynninen M, Bäcklund M (2017). Inhaled Xenon attenuates myocardial damage in comatose survivors of out-of-hospital cardiac arrest: the Xe-Hypotheca trial. J Am Coll Cardiol..

[41] Weber NC, Toma O, Wolter JI, Obal D, Müllenheim J, Preckel B (2005). The noble gas xenon induces pharmacological preconditioning in the rat heart in vivo via induction of PKC-ε and p38 MAPK. Br J Pharmacol.

[42] Hofland J, Ouattara A, Fellahi JL, Gruenewald M, Hazebroucq J, Ecoffey C (2017). Effect of xenon anesthesia compared to sevoflurane and total intravenous anesthesia for coronary artery bypass graft surgery on postoperative cardiac troponin release an international, multicenter, phase 3, single-blinded, randomized noninferiority trial. Anesthesiology.

[43] Laitio R, Hynninen M, Arola O, Virtanen S, Parkkola R, Saunavaara J (2016). Effect of inhaled xenon on cerebral white matter damage in comatose survivors of out-of-hospital cardiac arrest: a randomized clinical trial. JAMA - J Am Med Assoc..

[44] Fragata I, Alves M, Papoila AL, Nunes AP, Ferreira P, Canto-Moreira N (2017). Early prediction of delayed ischemia and functional outcome in acute subarachnoid hemorrhage: role of diffusion tensor imaging. Stroke.

[45] Chan AW, Tetzlaff JM, Gøtzsche PC, Altman DG, Mann H, Berlin JA, SPIRIT,  (2013). explanation and elaboration: guidance for protocols of clinical trials. BMJ..

[46] Hoh BL, Ko NU, Amin-Hanjani S, Hsiang-Yi Chou S, Cruz-Flores S, Dangayach NS, Derdeyn CP, Du R, Hänggi D, Hetts SW, Ifejika NL, Johnson R, Keigher KM, Leslie-Mazwi TM, Lucke-Wold B, Rabinstein AA, Robicsek SA, Stapleton CJ, Suarez JI, Tjoumakaris SI, … Welch BG. 2023 Guideline for the Management of Patients With Aneurysmal Subarachnoid Hemorrhage: A Guideline From the American Heart Association/American Stroke Association. Stroke. 2023;10.1161/STR.0000000000000436. Advance online publication. 10.1161/STR.0000000000000436.

[47] Grinnon ST, Miller K, Marler JR, Lu Y, Stout A, Odenkirchen J, Kunitz S. National Institute of Neurological Disorders and Stroke Common Data Element Project - approach and methods. Clin Trials (London, England). 2012;9(3):322–29. 10.1177/1740774512438980. 10.1177/1740774512438980 PMC3513359 22371630

[48] de Oliveira Manoel AL, van der Jagt M, Amin-Hanjani S, Bambakidis NC, Brophy GM, Bulsara K (2019). Common Data Elements for Unruptured Intracranial Aneurysms and Aneurysmal Subarachnoid Hemorrhage: Recommendations from the Working Group on Hospital Course and Acute Therapies—Proposal of a Multidisciplinary Research Group. Neurocrit Care.

[49] Jeurissen B, Leemans A, Tournier JD, Jones DK, Sijbers J (2013). Investigating the prevalence of complex fiber configurations in white matter tissue with diffusion magnetic resonance imaging. Hum Brain Mapp.

[50] Tournier JD, Calamante F, Gadian DG, Connelly A (2004). Direct estimation of the fiber orientation density function from diffusion-weighted MRI data using spherical deconvolution. Neuroimage.

[51] Tournier JD, Calamante F, Connelly A (2007). Robust determination of the fibre orientation distribution in diffusion MRI: Non-negativity constrained super-resolved spherical deconvolution. Neuroimage.

[52] Jeurissen B, Leemans A, Jones DK, Tournier JD, Sijbers J (2011). Probabilistic fiber tracking using the residual bootstrap with constrained spherical deconvolution. Hum Brain Mapp.

[53] Bullmore E, Sporns O. Complex brain networks: graph theoretical analysis of structural and functional systems. Nat Rev Neurosci. 2009;10:186–98. 10.1038/nrn2575. 10.1038/nrn2575 19190637

[54] Radlinska BA, Ghinani SA, Lyon P, Jolly D, Soucy JP, Minuk J (2009). Multimodal microglia imaging of fiber tracts in acute subcortical stroke. Ann Neurol.

[55] Rissanen E, Tuisku J, Vahlberg T, Sucksdorff M, Paavilainen T, Parkkola R (2018). Microglial activation, white matter tract damage, and disability in MS. Neurol Neuroimmunol Neuroinflamm.

[56] Rissanen E, Tuisku J, Rokka J, Paavilainen T, Parkkola R, Rinne JO (2014). In vivo detection of diffuse inflammation in secondary progressive multiple sclerosis using PET imaging and the radioligand11C-PK11195. J Nucl Med.

[57] Mathias S, Younes A, Kan CC, Orlow I, Joseph C, Kolesnick RN (1993). Activation of the sphingomyelin signaling pathway in intact EL4 cells and in a cell-free system by IL-1β. Science (80- ).

[58] Dickens AM, Borgan F, Laurikainen H, Lamichhane S, Marques T, Rönkkö T (2020). Links between central CB1-receptor availability and peripheral endocannabinoids in patients with first episode psychosis. NPJ Schizophr.

[59] Castillo S, Mattila I, Miettinen J, Orešič M, Hyötyläinen T (2011). Data analysis tool for comprehensive two-dimensional gas chromatography/time-of-flight mass spectrometry. Anal Chem.

[60] Nygren H, Seppänen-Laakso T, Castillo S, Hyötyläinen T, Orešič M. Liquid chromatography-mass spectrometry (LC-MS)-based lipidomics for studies of body fluids and tissues. Methods Mol Biol (Clifton, N.J.). 2011;708:247–57. 10.1007/978-1-61737-985-7_15. 10.1007/978-1-61737-985-7_15 21207295

[61] Pluskal T, Castillo S, Villar-Briones A, Orešič M (2010). MZmine 2: Modular framework for processing, visualizing, and analyzing mass spectrometry-based molecular profile data. BMC Bioinformatics.

[62] Harris PA, Taylor R, Thielke R, Payne J, Gonzalez N, Conde JG (2009). Research electronic data capture (REDCap)-a metadata-driven methodology and workflow process for providing translational research informatics support. J Biomed Inform.

[63] Harris PA, Taylor R, Minor BL, Elliott V, Fernandez M, O’Neal L (2019). The REDCap consortium: building an international community of software platform partners. J Biomed Inform.

[64] Daqing M, Lim T, Xu J, Tang H, Wan Y, Zhao H, Hossain M, Maxwell PH, Maze M (2009). Xenon preconditioning protects against renal ischemic-reperfusion injury via hif-1α activation. J Am Soc Nephrol.

[65] Kostandy BB (2012). The role of glutamate in neuronal ischemic injury: the role of spark in fire. Neurol Sci.

[66] Banks P, Franks NP, Dickinson R (2010). Competitive inhibition at the glycine site of the n-methyl-d-aspartate receptor mediates xenon neuroprotection against hypoxia-ischemia. Anesthesiology.

[67] Dinse A, Föhr KJ, Georgieff M, Beyer C, Bulling A, Weigt HU (2005). Xenon reduces glutamate-, AMPA-, and kainate-induced membrane currents in cortical neurones. Br J Anaesth.

[68] Gruss M, Bushell TJ, Bright DP, Lieb WR, Mathie A, Franks NP (2004). Two-Pore-Domain K+ Channels Are a Novel Target for the Anesthetic Gases Xenon, Nitrous Oxide, and Cyclopropane. Mol Pharmacol.

[69] Bantel C, Maze M, Trapp S (2009). Neuronal preconditioning by inhalational anesthetics: Evidence for the role of plasmalemmal adenosine triphosphate-sensitive potassium channels. Anesthesiology.

[70] Laitio RM, Kaisti KK, Låangsjö JW, Aalto S, Salmi E, Maksimow A (2007). Effects of xenon anesthesia on cerebral blood flow in humans: A positron emission tomography study. Anesthesiology.

[71] Laitio RM, Långsjö JW, Aalto S, Kaisti KK, Salmi E, Maksimow A (2009). The effects of xenon anesthesia on the relationship between cerebral glucose metabolism and blood flow in healthy subjects: a positron emission tomography study. Anesth Analg.

[72] Fragata I, Alves M, Papoila AL, Ferreira P, Nunes AP, Moreira NC, Canhão P. Prediction of clinical outcome in subacute subarachnoid hemorrhage using diffusion tensor imaging. J Neurosurg. 2018:1–9. Advance online publication. 10.3171/2017.10.JNS171793. 10.3171/2017.10.JNS171793 29652228

[73] Sener S, Van Hecke W, Feyen BFE, Van Der Steen G, Pullens P, Van De Hauwe L (2016). Diffusion Tensor Imaging: A Possible Biomarker in Severe Traumatic Brain Injury and Aneurysmal Subarachnoid Hemorrhage?. Neurosurgery.

[74] Yeo SS, Choi BY, Chang CH, Kim SH, Jung YJ, Jang SH (2012). Evidence of corticospinal tract injury at midbrain in patients with subarachnoid hemorrhage. Stroke.

[75] Jang SH, Choi BY, Kim SH, Chang CH, Jung YJ, Kwon HG (2014). Injury of the mammillothalamic tract in patients with subarachnoid haemorrhage: a retrospective diffusion tensor imaging study. BMJ Open.

[76] Kummer TT, Magnoni S, MacDonald CL, Dikranian K, Milner E, Sorrell J (2015). Experimental subarachnoid haemorrhage results in multifocal axonal injury. Brain.

[77] Wu Y, Peng J, Pang J, Sun X, Jiang Y (2017). Potential mechanisms of white matter injury in the acute phase of experimental subarachnoid haemorrhage. Brain.

[78] Song SK, Sun SW, Ramsbottom MJ, Chang C, Russell J, Cross AH (2002). Dysmyelination revealed through MRI as increased radial (but unchanged axial) diffusion of water. Neuroimage.

[79] Song SK, Sun SW, Ju WK, Lin SJ, Cross AH, Neufeld AH (2003). Diffusion tensor imaging detects and differentiates axon and myelin degeneration in mouse optic nerve after retinal ischemia. Neuroimage.

[80] Song SK, Yoshino J, Le TQ, Lin SJ, Sun SW, Cross AH (2005). Demyelination increases radial diffusivity in corpus callosum of mouse brain. Neuroimage.

[81] Hravnak M, Frangiskakis JM, Crago EA, Chang Y, Tanabe M, Gorcsan J (2009). Elevated cardiac troponin i and relationship to persistence of electrocardiographic and echocardiographic abnormalities after aneurysmal subarachnoid hemorrhage. Stroke.

[82] Deibert E, Barzilai B, Braverman AC, Edwards DF, Aiyagari V, Dacey R (2003). Clinical significance of elevated troponin I levels in patients with nontraumatic subarachnoid hemorrhage. J Neurosurg.

[83] Wartenberg KE, Schmidt JM, Claassen J, Temes RE, Frontera JA, Ostapkovich N (2006). Impact of medical complications on outcome after subarachnoid hemorrhage. Crit Care Med.

[84] Banki N, Kopelnik A, Tung P, Lawton MT, Gress D, Drew B (2006). Prospective analysis of prevalence, distribution, and rate of recovery of left ventricular systolic dysfunction in patients with subarachnoid hemorrhage. J Neurosurg.

[85] Naredi S, Lambert G, Friberg P, Zäll S, Edén E, Rydenhag B (2006). Sympathetic activation and inflammatory response in patients with subarachnoid haemorrhage. Intensive Care Med.

[86] Naredi S, Lambert G, Edén E, Zäll S, Runnerstam M, Rydenhag B (2000). Increased sympathetic nervous activity in patients with nontraumatic subarachnoid hemorrhage. Stroke.

[87] Lee VH, Oh JK, Mulvagh SL, Wijdicks EFM (2006). Mechanisms in neurogenic stress cardiomyopathy after aneurysmal subarachnoid hemorrhage. Neurocrit Care.

[88] Moussouttas M, Huynh TT, Khoury J, Lai EW, Dombrowski K, Pello S (2012). Cerebrospinal fluid catecholamine levels as predictors of outcome in subarachnoid hemorrhage. Cerebrovasc Dis.

[89] Tung P, Kopelnik A, Banki N, Ong K, Ko N, Lawton MT (2004). Predictors of Neurocardiogenic Injury after Subarachnoid Hemorrhage. Stroke.

[90] Ogura T, Satoh A, Ooigawa H, Sugiyama T, Takeda R, Fushihara G (2012). Characteristics and prognostic value of acute catecholamine surge in patients with aneurysmal subarachnoid hemorrhage. Neurol Res.

[91] Temes RE, Tessitore E, Schmidt JM, Naidech AM, Fernandez A, Ostapkovich ND (2010). Left ventricular dysfunction and cerebral infarction from vasospasm after subarachnoid hemorrhage. Neurocrit Care.

